# Moulage 2.0: Eine Querschnittsstudie zu einem 3D-gedruckten Hautmodell in der dermatologischen Lehre und Weiterbildung

**DOI:** 10.1007/s00105-025-05579-w

**Published:** 2025-09-11

**Authors:** Alexander Schneller, Hannah Wecker, Michael Hindelang, Sandra Schuh, Julia Welzel, Alexander Zink

**Affiliations:** 1 Klinik und Poliklinik für Dermatologie und Allergologie am Biederstein, Fakultät für Medizin, Technische Universität München, München, Deutschland; 2 Klinik für Dermatologie und Allergologie, Universitätsklinikum Augsburg, Sauerbruchstr. 6, 86179 Augsburg, Deutschland; 3 Pettenkofer School of Public Health München, München, Deutschland; 4 Institut für Medizinische Informationsverarbeitung Biometrie und Epidemiologie (IBE), Ludwigs-Maximilians-Universität München, München, Deutschland; 5 Institut für Digitale Medizin, Universitätsklinikum Augsburg, Augsburg, Deutschland

**Keywords:** Geschichte der Medizin, Dreidimensionale Bildgebung, 3D-Druck, Lehre in der Medizin, Anatomische Modelle, Moulagen, History of medicine, Imaging, three-dimensional, Printing, three-dimensional, Education, medical, Models, anatomic, Moulages

## Abstract

**Hintergrund und Ziel der Arbeit:**

Historische dreidimensionale Rekonstruktionen von Hautkrankheiten, „Moulagen“, waren früher in der medizinischen Ausbildung unverzichtbar und sind mit dem Aufkommen der Fotografie in den Hintergrund getreten. Dreidimensionale Merkmale, die durch zweidimensionale Fotografien nicht vermittelt werden können, sind in der Dermatologie unerlässlich für die korrekte Diagnose von Hautkrankheiten. Ziel dieser Studie war es, die Anwendung einer mit modernen 3D-Technologien erstellten Moulage in der dermatologischen Lehre und Weiterbildung zu erproben und ihren Nutzen bei der Effloreszenzlehre in der dermatologischen Aus- und Weiterbildung im Vergleich zu etablierten Lehrmedien zu evaluieren.

**Material und Methoden:**

Der 3D-Scan eines Gesichts wurde digital mit 5 dermatologischen Effloreszenzen, Plaque, Makula, Noduli, Cicatrices und Pusteln, zu einem virtuellen 3D-Modell ergänzt. Mithilfe von 3D-Druck wurde im Polyjet-Verfahren ein vollfarbiges 3D-Modell in Originalgröße aus einem flexiblen Photopolymer generiert. Dieses wurde im Rahmen einer Querschnittserhebung an einer deutschen Universitätshautklinik an 214 Medizinstudierenden des zweiten klinischen Studienjahrs, 22 WeiterbildungsassistentInnen und 9 FachärztInnen im Vergleich zu zweidimensionalen und dreidimensionalen virtuellen Abbildungen von Hautbefunden mittels auszufüllender Fragebögen evaluiert.

**Ergebnisse:**

Subjektiv glaubten die Teilnehmenden, Hauteffloreszenzen am schlechtesten anhand konventioneller Fotografien korrekt identifizieren zu können. Höhere Werte für die empfundene Diagnosesicherheit zeigten sich bei virtuellen 3D-Modellen, mit den höchsten Werten für die 3D-Moulage. Die Teilnehmenden befürworteten die Integration von modernen Moulagen in die medizinische Ausbildung und die potenzielle Verwendung von 3D-Modellen für chirurgisches Training. Vor allem Medizinstudierende hielten 3D-Moulagen für hilfreich beim Erlernen dermatologischer Effloreszenzen.

**Diskussion:**

Mit dieser Studie sollte der Nutzen von 3D-Technologien in der zeitgemäßen dermatologischen Lehre evaluiert werden. Konventioneller 2D-Fotografie fehlt die haptische Information, die für eine sichere Identifizierung von Effloreszenzen erforderlich ist. 3D-Technologien verbessern das Verständnis und die Sicherheit bei der Erkennung von Hautläsionen. Moderne 3D-Technologien bieten die Möglichkeit, durch multisensorielles Lernen die dermatologische Lehre zu bereichern.

## Abstract (en)

**Background and objective:**

Historical three-dimensional (3D) reconstructions of skin diseases, known as “moulages,” were once indispensable in medical education but diminished in importance with the advent of photography. However, 3D characteristics that cannot be conveyed through two-dimensional (2D) photographs are essential in dermatology for the accurate diagnosis of skin diseases. The aim of this study was to test the use of a moulage created with modern 3D technologies in dermatological education and training, and to evaluate its benefit for teaching primary skin lesions (efflorescences) compared to established teaching media.

**Materials and methods:**

A 3D scan of a face was digitally augmented with five dermatological primary lesions: plaque, macule, nodules, scars, and pustules. Using 3D printing polyjet technology, a full-color, 3D model in a flexible photopolymer material was generated in original size. This model was evaluated through a cross-sectional survey at a German university dermatology department, involving 214 medical students in the second clinical year, 22 dermatology residents, and 9 board-certified dermatologists. The model was compared to 2D and virtual 3D representations of skin findings using structured questionnaires completed by the participants.

**Results:**

Subjectively, participants believed that conventional photographs were the least effective means of correctly identifying skin eruptions. Higher scores for perceived diagnostic confidence were observed with virtual 3D models, with the highest scores achieved using the 3D moulage. Participants supported the integration of modern moulages into medical education and the potential use of 3D models for surgical training. Medical students in particular found 3D moulages helpful for learning dermatological efflorescences.

**Conclusion:**

This study aimed to evaluate the usefulness of 3D technologies in modern dermatological education. Conventional 2D photography lacks the haptic information required for confident identification of primary skin lesions. Furthermore, 3D technologies enhance understanding and improve confidence in recognizing skin lesions. Modern 3D technologies offer an opportunity to enrich dermatological education through multisensory learning.

## Hinführung zum Thema

NeuesteInnovationen in industriellen 3D-Verfahren ermöglichen erstmals die Wiedereinführung von dermatologischen 3D-Modellen „Moulagen“ in die dermatologische Lehre. Mithilfe eines so entstandenen Modells wurde der Nutzen von 3D-Modellen in der dermatologischen Lehre untersucht.

## Einführung

**Abb. 1:**
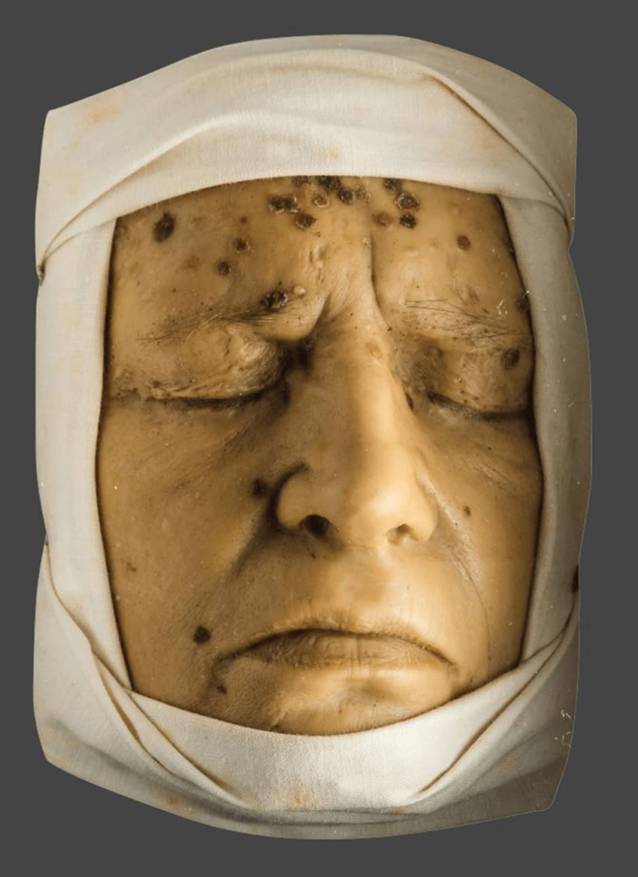
Moulage Frühsyphilis. (Foto: Alexander Burzik. Veröffentlichung mit freundl. Genehmigung von © Stiftung Deutsches Hygiene-Museum Dresden)

Bis zur ersten Hälfte des 20. Jahrhunderts waren dreidimensionale Rekonstruktionen von Hautkrankheiten, sogenannte „Moulagen“, in der medizinischen Lehre unverzichtbar (Abb. 1) und die Technik dahinter war ein gut gehütetes Geheimnis [1]. Viele europäische Kliniken beschäftigten „Mouleure“, seit dem 18. Jahrhundert wurden Moulagen von neu aufgenommenen Patienten angefertigt, wodurch umfangreiche Sammlungen entstanden [2]. Noch heute umfasst die Moulagensammlung der Dermatologischen Klinik des Universitätsspitals Zürich mehr als 1800 und die der Universität Bonn mehr als 1000 Exemplare [3, 4]. Mit der Einführung der vergleichsweise günstigen Farbfotografie in der Mitte des 20. Jahrhunderts wurde das arbeitsintensive Medium der Moulagen jedoch zunehmend aufgegeben [5]. Diese sind heute nur noch in medizinhistorischen Sammlungen zu bewundern, sofern sie nicht im Krieg zerstört oder zu Kerzenwachs eingeschmolzen wurden, wie die Sammlung in Sendai, Japan [6]. In der Dermatologie könnte sich diese Veränderung potenziell nachteilig auf die Lehre ausgewirkt haben, da haptische und dreidimensionale Merkmale für die Diagnose und erfolgreiche Behandlung vieler Hauterkrankungen entscheidend sind. Die korrekte Benennung von Hauteffloreszenzen ist in der Lehre ein Hauptlernziel und Voraussetzung für das pathophysiologische Verständnis von Hauterkrankungen.

In der Dermatologie werden mit Begriffen wie „Papel“ oder „Plaque“ dreidimensionale Merkmale von Hautläsionen beschrieben, die sich nicht immer aus fotografischen Abbildungen ableiten lassen. Dennoch stützt sich die dermatologische Lehre, wenn kein klinischer Unterricht an PatientInnen möglich ist, heute fast ausschließlich auf zweidimensionale Fotografien in Lehrbüchern, Vorlesungen oder Online-Kursen. Ob diese Praxis des weitgehenden Fehlens haptischer Lehrmaterialien eine Einschränkung in der dermatologischen Lehre darstellt, wenn diese nicht direkt an PatientInnen durchgeführt werden kann, ist bisher nicht entsprechend untersucht.

Die bei der Herstellung von Moulagen verwendete Methode der Reproduktion von Hautbefunden durch Negativabformung der Patienten mit Gips und Herstellung eines Wachspositivs, das anschließend bemalt wurde, ist aus verschiedenen Gründen nicht mehr praktikabel [7]: Erstens kann der Kontakt mit Fremdstoffen bei vielen Hautbefunden kontraindiziert sein. Zweitens ist der Prozess des Abformens, Gießens und Färbens zeitaufwendig und erfordert ein hohes Maß an handwerklichem Geschick, das mit dem Niedergang der Moulagen in Vergessenheit geraten ist. Zudem stellen die empfindlichen Wachsmoulagen auch eine Herausforderung dar in der hitzegeschützten Lagerung, insbesondere in Zeiten steigender Temperaturen, und benötigen große Lagerkapazitäten.

3D-Technologien, v. a. der 3D-Druck, gewinnen in der modernen medizinischen Lehre und Behandlung immer mehr an Bedeutung. Vor allem in handwerklich fordernden Fachdisziplinen, wie der Kinderherzchirurgie, entwickelt sich der Einsatz von 3D-Technologien rasant und hat durch die zusätzliche Dimension ein hohes Potenzial für die Verbesserung der medizinischen Lehre [8]. In der Dermatologie gibt es bereits innovative Versuche, die haptische Dimensionen wieder in die dermatologische Lehre einzubringen, z. B. indem an gesunden PatientInnen Silikonnachbildungen von Hautkrankheiten angebracht wurden, die dann von Studierenden untersucht werden konnten [9].

Der einzige den AutorInnen bekannte Versuch, die vergessene Kunst der Moulagen durch den Einsatz moderner 3D-Drucktechnologien in der dermatologischen Lehre wiederzubeleben, bestand aus einer kleinen Silikonmatte, die mit Farben und Erhebungen Effloreszenzen, wie Makula, Plaque, Nodus und andere, abstrakt imitierte [10]. Bei dieser Intervention kam keine 3D-Scanning-Technologie zum Einsatz und der 3D-Druck diente zur Erstellung einer Negativform, die ein arbeitsintensives manuelles Abformen und Einfärben erforderte. Die teilnehmenden 222 Medizinstudierenden berichteten jedoch von einem positiven Einfluss auf ihr Lernen aufgrund des neuen Lehrmittels, was den positiven Einfluss des haptischen Unterrichts in der Dermatologie und den Bedarf an weiterer Forschung unterstreicht.

Jüngste Fortschritte in der 3D-Druck- und 3D-Scantechnologie ermöglichen es nun erstmals, lebensechte Reproduktionen von Hautbefunden herzustellen. Die moderne Neuinterpretation von Moulagen, ohne Kompromisse bei Detailgenauigkeit und Realismus einzugehen, und ihre Evaluation in der medizinischen Ausbildung ist ein Ansatz, der von Wissenschaftlern gefordert, aber noch nicht in die Praxis umgesetzt wurde [11].

Moulagen konnten in der Vergangenheit erfolgreich die komplexen haptischen Charakteristika verschiedener Hautkrankheiten vermitteln. Um den Limitationen der in der dermatologischen Lehre überwiegend verwendeten 2D-Fotografien zu begegnen, sollte mit dieser Studie die Herstellung einer mit modernen 3D-Technologien geschaffenen Moulage erstmalig erprobt und deren potenzieller Nutzen für die Vermittlung von Effloreszenzen im Vergleich zu zweidimensionalen Lehrmedien in der dermatologischen Lehre und Weiterbildung evaluiert werden.

## Material und Methoden

Bei der Herstellung der modernen Moulage wurden die Charakteristika des historischen Vorbilds, insbesondere Dreidimensionalität, Fotorealismus und lebensechte Haptik, zugrunde gelegt. Da viele historische Moulagen Nachbildungen von PatientInnengesichtern darstellen, wurde der 3D-Scan des Kopfes eines Probanden als Basismodell für die moderne Moulage verwendet. Die Entscheidung hierfür fiel zum einen mangels historischer Moulage an den beteiligten Kliniken. Zum anderen sollte in einem Modell eine Vielzahl von Effloreszenzen abgebildet werden, wobei historische Moulagen meistens nur eine einzelne Dermatose abbilden.

**Abb. 2:**
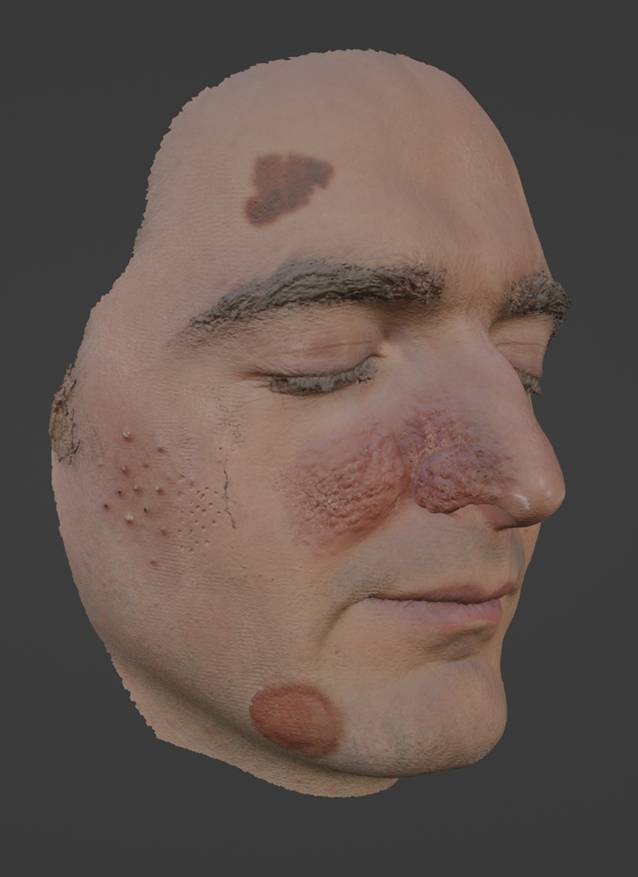
Virtuelles 3D-Modell der generierten Moulage

**Abb. 3:**
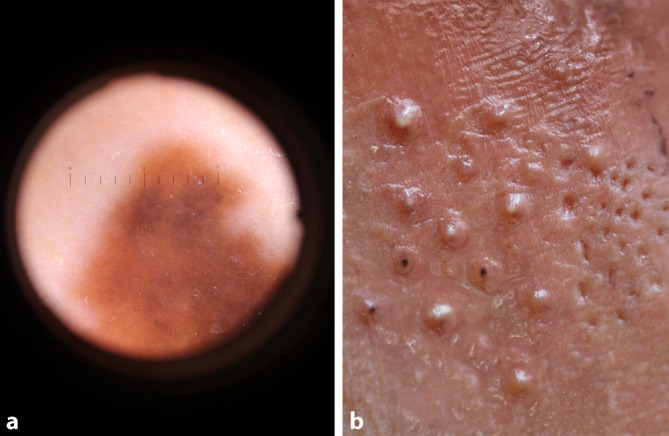
**a** Reproduktion einer Lentigo maligna auf der gedruckten Moulage, Blick durch das Dermatoskop. **b** Pusteln, Komedonen und Narben auf der gedruckten Moulage

**Abb. 4:**
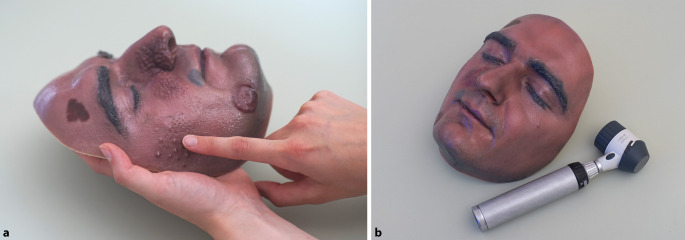
**a** Die gedruckte Moulage kann haptisch erfasst werden. **b** Die gedruckte Moulage ist mit dem Dermatoskop untersuchbar

Mit einem handgeführten 3D-Scanner (Space Spider, Artec 3D, Senningerberg, Luxemburg) wurde das Gesicht des Autors in ein 3D-Modell überführt. Hierbei kam eine Strukturlichttechnologie zum Einsatz, die ohne die Verwendung von Lasern keine Gefahr für das Augenlicht von Probanden und Bediener darstellt. Das damit aufgenommene Gesicht war zunächst frei von Hautkrankheiten. Entsprechend ihrer ursprünglichen Nutzung wurde der größte Benefit von Moulagen in der Vermittlung grundlegender dermatologischer Fähigkeiten wie der Effloreszenzenlehre erwartet. Einfache dermatologische Befunde wurden auf der rechten Seite unseres Modells hinzugefügt, die linke Seite blieb frei von Pathologien (Abb. 2). Dies geschah mit der leistungsstarken 3D-Modellierungssoftware Blender (Blender Foundation, Amsterdam, Niederlande; [12]). Zur Veranschaulichung einer Makula wurde eine Lentigo maligna auf der Stirn angebracht (Abb. 3a mit Blick durch das Dermatoskop), für eine Plaque eine Tinea am Kinn, für Pusteln und Narben eine Acne vulgaris an der Wange (Abb. 3b), zur Veranschaulichung von Noduli und einem Erythem wurde eine Rosazea erzeugt, die sich über Nase und Wange erstreckte, und ein bereits vorhandener dermaler Naevus im Bereich des linken Jochbeins des Autors wurde zur Veranschaulichung einer Papel belassen (Abb. 4a, b). Mit diesen Schritten entstand ein virtuelles 3D-Modell, das mithilfe eines PCs betrachtet werden konnte und als Grundlage für den 3D-Druck diente.

Das 3D-Modell wurde in Lebensgröße auf einem industriellen 3D-Drucker (J850 Digital Anatomy, Stratasys, Rechovot, Israel) unter Verwendung der Polyjet-Technologie gedruckt, die fotorealistische dreidimensionale Drucke in einer Vielzahl von Materialien ermöglichte. Da die menschliche Haut eine Härte von etwa 21 auf der Shore-A-Skala aufweist [13], wurde das weichste für Polyjet-Drucker verfügbare Photopolymermaterial mit einer Härte von etwa 30 Shore gewählt [14] und das Modell massiv gedruckt. Sowohl der animierte 3D-Scan als auch die gedruckte Moulage wurden dann wie oben beschrieben in der Untersuchung eingesetzt.

Im Sommersemester 2023 wurde von April bis Juli an einer dermatologischen Universitätsklinik in München eine Querschnittstudie zur Erprobung und Evaluation der Moulage in der dermatologischen Lehre und Weiterbildung durchgeführt.

Insgesamt wurden 245 Teilnehmende für die Studie rekrutiert. Die meisten von ihnen waren Medizinstudierende (214) im zweiten klinischen Studienjahr (7. und 8. Semester), die im Rahmen ihres dermatologischen Curriculums an einer Lehrveranstaltung zur Effloreszenzenlehre teilnahmen. Zusätzlich wurden während einer der routinemäßig stattfindenden täglichen Mittagsvisiten ärztliche KollegInnen als Schlüsselpersonen der universitären Lehre und Durchführende der konventionellen Lehreinheiten zur Effloreszenzlehre zu ihrer Einschätzung des 3D-Modells befragt. Dies waren 22 AssistenzärztInnen, von denen die meisten seit 0–3 Jahren praktizierten, sowie 9 FachärztInnen, von denen zwei Drittel mehr als 4 Jahre und ein Drittel mehr als 10 Jahre Erfahrung in ihrer Position hatten (Tab. 1).

**Tab. 1:** Probandencharakteristiken (neu)

Item	Skala	Gesamt	Medizinstudierende	WeiterbildungsassistentInnen	FachärztInnen
1. Anhand der gezeigten konventionellen Fotografien lassen sich Effloreszenzen sicher beschreiben: Median (IQR)	1 (nicht sicher)–10 (sicher)	4,00 [3,00, 6,00]	4,00 [3,00, 6,00]	5,00 [2,25, 7,00]	6,00 [5,00, 7,00]
2. Anhand des gezeigten 3D-Scans lassen sich Effloreszenzen sicher beschreiben: Median (IQR)	–	8,00 [7,00, 9,00]	8,00 [6,75, 9,00]	8,00 [7,00, 8,00]	8,00 [8,00, 8,00]
	Fehlend	2	2	–	–
3. Anhand der gezeigten physischen Moulage lassen sich Effloreszenzen sicher beschreiben: Median (IQR)	–	9,00 [9,00, 10,00]	9,00 [9,00, 10,00]	9,00 [8,00, 9,75]	9,00 [9,00, 10,00]

*IQR* Interquartilsbereich

Der Befragung ging eine etwa 15-minütige Einführung zu dermatologischen Effloreszenzen mithilfe von konventionellen, zweidimensionalen Fotoabbildungen von Hauteffloreszenzen voraus. In dieser Präsentation, die alle Probanden erhielten, wurden auch die Effloreszenzen auf dem virtuellen 3D-Modell der Moulage gezeigt, das mit 3D-Scan entstanden war und die Grundlage für den 3D-Druck darstellte. Anschließend hatten die Teilnehmenden die Gelegenheit, die physische 3D-Moulage für einige Minuten in Augenschein zu nehmen und zu betasten, bevor die Fragebogen ausgefüllt wurden. Im Rahmen dieser erstmaligen Anwendungsprobung einer hochrealistischen 3D-Moulage in Lehre und Weiterbildung wurde das Stimmungsbild der Teilnehmenden als Querschnittsstudie zu einem Zeitpunkt abgefragt. Ein Follow-up erfolgte nicht.

**Abb. 5:**
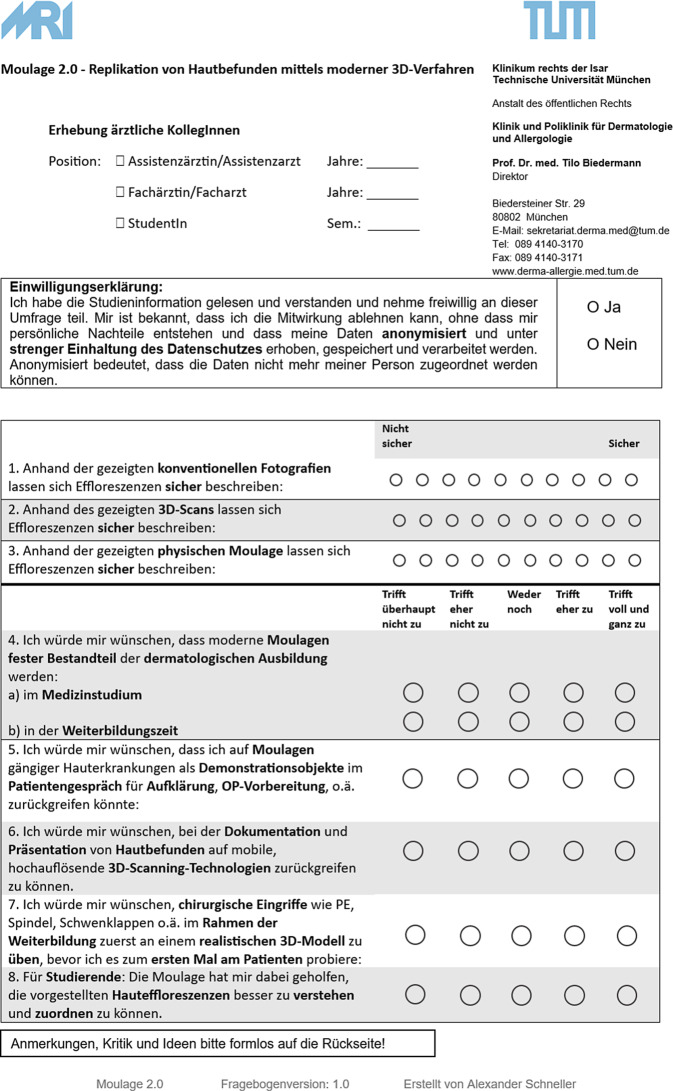
Fragebogen der Querschnittserhebung (neu)

Unsere primären Ergebnisse konzentrierten sich auf die Erhebung der Erfahrungen und Eindrücke von Studierenden und KlinikerInnen in Bezug auf die Moulage als Lehrmittel im Sinne eines „proof of concept“ hinsichtlich der Verwendung von modernen 3D-Simulatoren in Lehre und Weiterbildung. Die Einschätzung der dreidimensionalen, fotorealistischen und lebensechten haptischen Eigenschaften der Moulage war dabei von besonderem Interesse, und es wurde abgefragt, wie sicher sich die Befragten hinsichtlich der korrekten Beschreibung von Effloreszenzen mithilfe von Fotos, virtuellem Modell und 3D-Moulage fühlten. Den Teilnehmenden wurde ein einseitiger Fragebogen mit 9 Items (Abb. 5) ausgehändigt, darunter 6 Items mit einer 5‑Punkte-Likert-Skala und 3 Items mit einer 10-Punkte-Likert-Skala, die darauf abzielten, die Meinungen der Teilnehmenden zu der Moulage detailliert zu erfassen.

Neben Angaben zum Studienstand der Studierenden bzw. Weiterbildungsstand der ÄrztInnen sollte auch ein Stimmungsbild bezüglich potenzieller zukünftiger Anwendungsgebiete von 3D-Simulatoren eingefangen werden, um ggf. künftige 3D-Simulatoren für diese Anwendungszwecke zu generieren. In diesem Zusammenhang wurden die Anwendung bei PatientInnenaufklärung, die Planung von Eingriffen und, da das weiche Material theoretisch auch Inzision und Naht erlauben würde, die Simulation chirurgischer Fertigkeiten als mögliche Anwendungsfelder identifiziert und evaluiert. Darüber hinaus wurde bezugnehmend auf das virtuelle, im 3D-Scan erstellte und modifizierte Modell, repräsentativ die Einschätzung zu 3D-Scanning von Hautbefunden in der klinischen Routine erhoben. Die letzte Frage, ob Moulagen hilfreich für das Verständnis und die Identifizierung von Hautläsionen sind, wurde nur den Studierenden gestellt. Zusätzlich wurden alle Studierenden gefragt, ob sie schon einmal mit Moulagen in Berührung gekommen waren. Die TeilnehmerInnen wurden auch aufgefordert, Kritik, Kommentare und Anregungen formlos zu notieren.

Das Feedback aus den Fragebögen wurde deskriptiv ausgewertet. Dabei wurden alle kategorialen Daten oder Skalen, die Teil des Fragebogens waren, berücksichtigt. Fehlende Daten oder nicht beantwortete Fragen wurden von der endgültigen Analyse ausgeschlossen. Die Daten finden sich im Anhang (Tab. 2). Diese wurden deskriptiv beschrieben, indem absolute und relative Häufigkeiten für kategoriale und ordinale Variablen sowie Median und Interquartilsbereich (IQR) für kontinuierliche und ordinale Variablen auf einer 10-Punkte-Likert-Skala angegeben wurden. Fehlende Daten oder Nichtbeantwortung wurden für jede Variable angegeben. Die Analysen wurden getrennt nach dem aktuellen beruflichen Status der TeilnehmerInnen (Medizinstudierende, AssistenzärztInnen, FachärztInnen) durchgeführt. Die Jahre der Berufserfahrung der befragten ÄrztInnen wurden in 3 Gruppen eingeteilt: „0–3 Jahre“, „4–9 Jahre“ und „> 10 Jahre“. Die statistischen Analysen wurden mit R Version 4.2.1 durchgeführt [15].

## Ergebnisse

Auf die Frage, ob sie schon einmal mit Moulagen in Berührung gekommen seien, gaben 97,6 % der Studierenden an, vor der Beschäftigung mit diesem neuen 3D-Modell keine Kenntnisse über die Existenz von Moulagen gehabt zu haben.

**Abb. 6:**
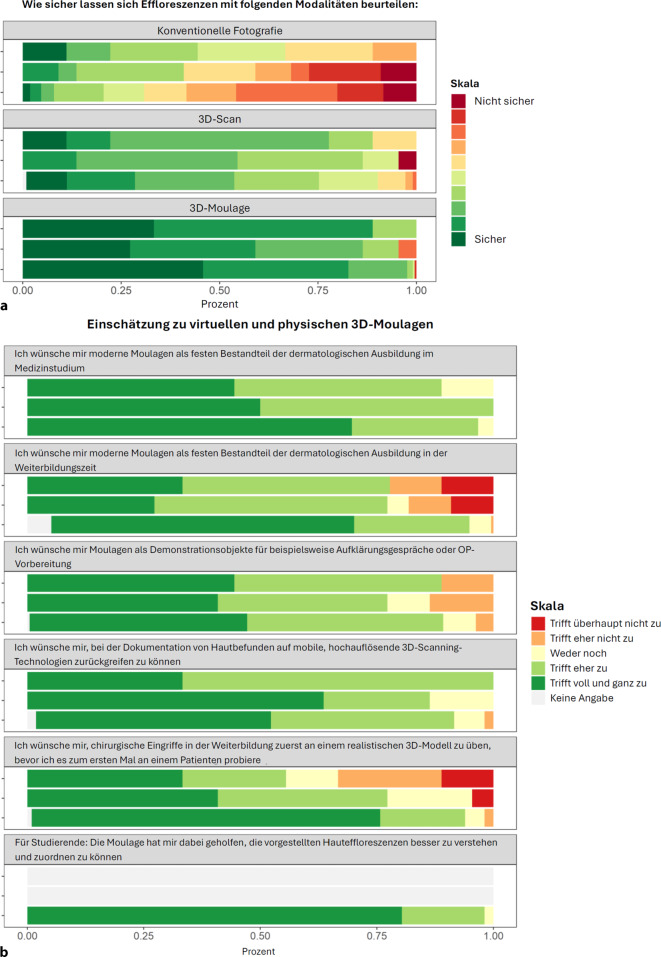
**a** Beurteilung der Effloreszenzen mit verschiedenen Modalitäten. **b** Einschätzung der virtuellen und physischen 3D-Moulagen

Die Teilnehmenden gaben an, dass sie eher nicht in der Lage waren, Effloreszenzen anhand herkömmlicher Fotos zuverlässig zu beschreiben (Median 4,00 [IQR: 3,00, 6,00]; Abb. 6a; Tab. 1), wobei Medizinstudierende und AssistenzärztInnen in ihrer Beschreibung weniger sicher waren als FachärztInnen. Anhand der mit 3D-Scans geschaffenen virtuellen Modelle würde den Befragten eine zuverlässigere Beschreibung gelingen (8,00 [7,00, 9,00]), und die meisten TeilnehmerInnen wären bei der Verwendung physischer moderner 3D-Moulagen noch sicherer (9,00 [9,00, 10,00]).

Die Integration von Moulagen in die humanmedizinische Lehre wurde von allen TeilnehmerInnen positiv bewertet: 96,7 % der Studierenden, 100 % der AssistenzärztInnen und 88,8 % der FachärztInnen stimmten teilweise oder voll und ganz zu, kein/e einzige/r der TeilnehmerInnen stimmte ganz oder teilweise nicht zu (Abb. 6b).

Auch bei der Frage, ob Moulagen zukünftig einen integralen Bestandteil der Facharztweiterbildung darstellen sollten, sahen die Medizinstudierenden einen großen Bedarf (94,6 % stimmten teilweise oder voll und ganz zu), während dies nur 77,3 % der AssistenzärztInnen und 77,7 % der FachärztInnen taten. Allerdings gaben 9,1 % der AssistenzärztInnen und 11,1 % der FachärztInnen an, dass sie hier definitiv keinen Bedarf sehen.

88,1 % der Befragten stimmten voll und ganz oder teilweise zu, Moulagen gerne für die Kommunikation mit PatientInnen oder die Operationsvorbereitung nutzen zu wollen, während 13,6 % der AssistenzärztInnen und 11,1 % der FachärztInnen hier eher abgeneigt waren. Für die Dokumentation und Präsentation von Hautbefunden stimmten 91,3 % der Teilnehmenden voll und ganz oder teilweise zu, dass sie sich hochauflösende 3D-Scan-Technologien wünschen würden.

Was die potenzielle Verwendung von zukünftigen 3D-Modellen zum Üben von chirurgischen Eingriffen vor der ersten Durchführung am Patienten/an der Patientin betrifft, so stimmten 93,3 % der Medizinstudierenden (mehr als die Hälfte von ihnen in den ersten 3 Jahren ihres Studiums) teilweise oder voll und ganz zu, dass sie sich diese Möglichkeit wünschen würden. Während der Anteil der FachärztInnen, die teilweise oder voll und ganz zustimmten, bei nur 55,5 % lag, und 33,3 % angaben, dass dies teilweise oder überhaupt nicht zutreffen würde.

98,2 % der Studierenden stimmten voll und ganz sowie teilweise zu, dass Moulagen ihnen helfen würden, die vorgestellten Effloreszenzen verstehen und identifizieren zu können.

Von den 15 abgegebenen Freitextkommentaren kamen 13 von Studierenden und 2 von WeiterbildungsassistentInnen. Eine*r der ÄrztInnen notierte, dass es sich insbesondere bei seltenen Dermatosen lohnen könne, originale Wachsmoulagen durch 3D-Scan zu konservieren. Die Studierenden erwähnten unter anderem die Vorteile der Moulage beim Üben von chirurgischen Eingriffen vor einer Operation, die Bedeutung bei Dokumentation und dem Unterricht von seltenen Erkrankungen, die nicht von allen Studierenden bei jedem Klinikaufenthalt zu Lehrzwecken gesehen werden können, sowie die Feststellung, dass insbesondere in Universitätskliniken sich Dermatosen oft ambulant vorbehandelt präsentieren würden und daher oft nicht so klar zuordenbar sind wie bei der Erstdiagnose. Es wurde auch erwähnt, dass das 3D-Modell ein Vorteil ist, wenn es um die Präsentation von Geschlechtskrankheiten geht, bei denen sich die Patienten unwohl fühlen könnten, vor vielen Studierenden exponiert zu sein und dass bei lebensechten Moulagen kein Risiko bestünde, sich beim Kontakt mit einem solchen Patienten/einer solchen Patientin anzustecken. Auch wurde als Stärke der Moulagen aufgeführt, dass die vorhandenen, aber langsam zerfallenden Moulagen aus Wachs reproduziert werden können. Schließlich wurde darauf hingewiesen, dass Moulagen mit verschiedenen Hauttönen nicht vergessen werden dürfen, da die meisten dermatologischen Lehrbücher Krankheiten mit kaukasischen PatientInnen abbilden, während dies oft nicht der ethnographischen Realität unserer PatientInnen entspricht.

## Diskussion

Ziel dieser Studie war es, ein Stimmungsbild hinsichtlich des potenziellen Nutzens moderner 3D-Moulagen in der dermatologischen Lehre und Weiterbildung durch Befragung von Medizinstudierenden und in der Dermatologie tätigen ÄrztInnen zu erheben. Die vorgestellten 3D-Technologien, durch die das traditionelle Lehrmedium Moulage für die zeitgemäße dermatologische Lehre transformiert wurde, erfuhren Zustimmung über alle Gruppen hinweg, wobei Medizinstudierende den Nutzen der Moulagen positiver bewerteten als die teilnehmenden ÄrztInnen. Die Teilnehmenden sprachen sowohl 3D-Moulagen als auch 3D-Scanning-Technologien Potenzial für die dermatologische Lehre sowie klinische Praxis zu.

Auch andere Erhebungen beschäftigen sich mit den Grenzen zweidimensionaler Fotos in der dermatologischen Ausbildung [5]. Indem den Teilnehmenden Hautbefunde in verschiedenen Realitätsgraden präsentiert wurden, nämlich zweidimensionale Bilder, dreidimensionale virtuelle Modelle und die 3D-gedruckte Moulage in Originalgröße, zeigte sich in dieser Untersuchung, dass die Probanden sich in der Beschreibung von Effloreszenzen signifikant sicherer fühlten, je lebensechter das Medium war. Dies deckt sich mit Studien, die die Wirksamkeit von taktilen Lehrmethoden in der medizinischen Ausbildung untersuchen [8, 10].

Mit zunehmender dermatologischer Erfahrung scheint es einfacher zu werden, eine Pathologie anhand eines zweidimensionalen Bildes zu bestimmen, obwohl in dieser Studie Fotografien auch von FachärztInnen als eine der 3D-Replikation unterlegene Quelle für die Identifizierung von Hautläsionen eingestuft wurden. Dies ist insofern bemerkenswert, wenn man bedenkt, dass die dermatologische Lehre und die Dokumentation von Hautläsionen heutzutage weltweit überwiegend auf zweidimensionalen Fotografien beruhen.

Die höheren Zustimmungsraten nach Hinzunehmen der dritten Dimension, beispielsweise bei den gezeigten 3D-Scans einer Hautläsion, stützt die Hypothese, dass konventionelle Fotografien möglicherweise zu wenig Informationen vermitteln, um dem Betrachter/der Betrachterin auch über die haptische Dimension des Befunds zu einer korrekten Diagnose zu verhelfen. Bei nochmals höheren Umfragewerten nach manueller Untersuchung der physischen Moulage durch die Teilnehmenden verstärkte sich diese Beobachtung. Dreidimensionale Moulagen sind potenziell die beste Möglichkeit, um Hauteffloreszenzen zu lehren, abgesehen vermutlich vom Kontakt mit einem echten Patienten/einer echten Patientin.

Aktuell existieren kaum hochrealistische 3D-Simulatoren für die dermatologische und dermatochirurgische Lehre und Weiterbildung. Der potenzielle Nutzen solcher modernen Moulagen in der medizinischen Ausbildung und beim Üben von chirurgischen Eingriffen wurde insbesondere von Medizinstudierenden positiv bewertet. Assistenz- und FachärztInnen äußerten sich verhaltener zur Integration von Moulagen in chirurgisches Training und Facharztausbildung, was möglicherweise darauf zurückzuführen ist, dass sie während ihrer eigenen Ausbildung auch ohne die Hilfe von 3D-Technologien erfolgreich waren.

Fast keiner der Studierenden wusste vor unserer Intervention von der Existenz von Moulagen. Dies könnte bedeuten, dass traditionelle Moulagen in der heutigen medizinischen Ausbildung keine sichtbare Rolle spielen und dass die in dieser Studie beobachteten Effekte nicht durch frühere Erfahrungen mit Moulagen verzerrt sind. Während historische Wachsmoulagen den Vorteil bieten, dass diese jederzeit verfügbar sind und eine Standardisierung der Lehre bei Schonung realer PatientInnen erlauben, ist dies nur an Zentren mit bestehenden Moulagensammlungen möglich, sollte es der Erhaltungszustand erlauben.

Moderne Moulagen können die Grenzen der momentan fast ausschließlich auf zweidimensionalen Abbildungen beruhenden Lehre erweitern. Das positive Feedback der Studierenden unterstreicht ihren Stellenwert in der medizinischen Grundausbildung und spiegelt Trends in der medizinischen Ausbildung wider [8, 10], während die auch kritische Einschätzung der KlinikerInnen auf ihre Komplexität bei der Einführung in der Fortbildung hinweist [9]. Zudem könnten moderne 3D-Moulagen dabei helfen, die bestehenden Bestände an historischen Wachsmoulagen zu digitalisieren und zu erhalten.

Neben der Dermatologie besitzen 3D-Technologien auch für andere Fachgebiete Potenzial, in denen praktische Fähigkeiten und eine genaue Auffassungsgabe erforderlich ist [8]. Eine mögliche Umsetzung hängt jedoch von den Ressourcen, der Gestaltung des Lehrplans und der Unterstützung durch die Lehrpersonen ab [16].

Die Limitationen dieser Studie beruhen einerseits auf der geringen Anzahl der teilnehmenden ÄrztInnen und des monozentrischen Studiendesigns. Somit lassen die Ergebnisse keine umfassenden Rückschlüsse zu, die positiven Ergebnisse dieser Studie zeigen jedoch das Potenzial von 3D-Technologien in der dermatologischen Ausbildung auf und begründen damit die Notwendigkeit weiterführender Untersuchungen. Darüber hinaus basieren die Ergebnisse auf den Angaben der Teilnehmenden selbst, was zu einer möglichen Verzerrung führen kann. Die Präsentation von 3D-Scans und Moulagen vor dem Ausfüllen des Fragebogens könnte zudem die Ergebnisse verzerrt haben. Außerdem erfordert die Erstellung von 3D-Scans und 3D-Moulagen weitreichende Softwarekenntnisse und ein entsprechendes Budget, wenn diese Techniken in die reguläre dermatologische Lehre klinische Routine integriert werden sollen. Letztlich steht die verwendete 3D-Druck-Technologie noch am Anfang ihrer Entwicklung. Auch wenn dies unseres Wissens die erste Moulage ist, die damit hergestellt wurde, besteht noch Verbesserungsbedarf, um bspw. mithilfe einer höheren räumlichen Auflösung das realistische Dermatoskopieren zu ermöglichen.

Zusammenfassend bietet in Anbetracht der zunehmenden Bedeutung von 3D-Technologien in der Medizin, des technischen Fortschritts und der positiven Ergebnisse unserer Beobachtungen die Wiedereinführung moderner 3D-Moulagen in allen Phasen der dermatologischen Ausbildung die Möglichkeit, in einem zunehmend theoretischen Medizinstudium Kompetenzen durch immersives Lernen zu erwerben.

Mit diesen neuen technischen Möglichkeiten könnte zudem die traditionelle zweidimensionale Fotografie bei der Dokumentation und Präsentation von Hautpathologien in Zukunft ergänzt werden, wie es bereits mit der Nutzung von Ganzkörperscannern bei der Hautkrebsvorsorge in manchen Hautkliniken erprobt wird. Darüber hinaus wäre es für die Dokumentation, Nachsorge, Forschung und Lehre von Vorteil, wenn hochauflösende 3D-Scans in die klinische Routine integriert würden.

## Fazit für die Praxis

- Mithilfe von 3D-Technologien lassen sich moderne Moulagen herstellen, die für die Lehre und Weiterbildung in der Dermatologie genutzt werden können.
- Diese stoßen auf Zuspruch bei Studierenden und Lehrenden und könnten einen Mehrwert in der Vermittlung von Hautkrankheiten bieten.
- Auch existierende historische Moulagen könnten gescannt bzw. gedruckt und so wieder nutzbar gemacht werden.

## Data Availability

Die dieser Studie zugrunde liegenden Daten können bei begründetem Interesse bei dem Erstautor dieser Publikation eingesehen werden.

## Anhang

**Tab. 2:** Antworten der Befragung (neu)

Item	Skala	Gesamt	Medizinstudierende	WeiterbildungsassistentInnen	FachärztInnen
4a. Ich würde mir wünschen, dass moderne Moulagen fester Bestandteil der dermatologischen Ausbildung im Medizinstudium werden: *n* (%)	Trifft überhaupt nicht zu	0 (0,0)	0 (0,0)	0 (0,0)	0 (0,0)
	Trifft eher nicht zu	0 (0,0)	0 (0,0)	0 (0,0)	0 (0,0)
	Weder noch	8 (3,3)	7 (3,3)	0 (0,0)	1 (11,1)
	Trifft eher zu	73 (29,8)	58 (27,1)	11 (50,0)	4 (44,4)
	Trifft voll und ganz zu	164 (66,9)	149 (69,6)	11 (50,0)	4 (44,4)
4b. Ich würde mir wünschen, dass moderne Moulagen fester Bestandteil der dermatologischen Ausbildung in der Weiterbildung werden: *n* (%)	Trifft überhaupt nicht zu	3 (1,3)	0 (0,0)	2 (9,1)	1 (11,1)
	Trifft eher nicht zu	4 (1,7)	1 (0,5)	2 (9,1)	1 (11,1)
	Weder noch	11 (4,7)	10 (4,9)	1 (4,5)	0 (0,0)
	Trifft eher zu	68 (29,1)	53 (26,1)	11 (50,0)	4 (44,4)
	Trifft voll und ganz zu	148 (63,2)	139 (68,5)	6 (27,3)	3 (33,3)
	Fehlend	11	11	–	–
5. Ich würde mir wünschen, dass ich auf Moulagen gängiger Hauterkrankungen als Demonstrationsobjekte im Patientengespräch für Aufklärung, Operationsvorbereitung, o. ä. zurückgreifen könnte: *n* (%)	Trifft überhaupt nicht zu	0 (0,0)	0 (0,0)	0 (0,0)	0 (0,0)
	Trifft eher nicht zu	12 (4,9)	8 (3,8)	3 (13,6)	1 (11,1)
	Weder noch	17 (7,0)	15 (7,0)	2 (9,1)	0 (0,0)
	Trifft eher zu	102 (41,8)	90 (42,3)	8 (36,4)	4 (44,4)
	Trifft voll und ganz zu	113 (46,3)	100 (46,9)	9 (40,9)	4 (44,4)
	Fehlend	1	1	–	–
6. Ich würde mir wünschen, bei der Dokumentation und Präsentation von Hautbefunden auf mobile, hochauflösende 3D-Scanning-Technologien zurückgreifen zu können: *n* (%)	Trifft überhaupt nicht zu	0 (0,0)	0 (0,0)	0 (0,0)	0 (0,0)
	Trifft eher nicht zu	4 (1,7)	4 (1,9)	0 (0,0)	0 (0,0)
	Weder noch	17 (7,1)	14 (6,7)	3 (13,6)	0 (0,0)
	Trifft eher zu	95 (39,4)	84 (40,0)	5 (22,7)	6 (66,7)
	Trifft voll und ganz zu	125 (51,9)	108 (51,4)	14 (63,6)	3 (33,3)
	Fehlend	4	4	–	–
7. Ich würde mir wünschen, chirurgische Eingriffe wie Probeexzision, Spindel, Schwenklappen o. ä. im Rahmen der Weiterbildung zuerst an einem realistischen 3D-Modell zu üben, bevor ich es zum ersten Mal am Patienten probiere: *n* (%)	Trifft überhaupt nicht zu	2 (0,8)	0 (0,0)	1 (4,5)	1 (11,1)
	Trifft eher nicht zu	6 (2,5)	4 (1,9)	0 (0,0)	2 (22,2)
	Weder noch	14 (5,8)	9 (4,2)	4 (18,2)	1 (11,1)
	Trifft eher zu	49 (20,2)	39 (18,4)	8 (36,4)	2 (22,2)
	Trifft voll und ganz zu	172 (70,8)	160 (75,5)	9 (40,9)	3 (33,3)
	Fehlend	2	2	–	–
8. Für Studierende: Die Moulage hat mir dabei geholfen, die vorgestellten Hauteffloreszenzen besser zu verstehen und zuordnen zu können: *n* (%)	Trifft überhaupt nicht zu	0 (0,0)	0 (0,0)	–	–
	Trifft eher nicht zu	0 (0,0)	0 (0,0)	–	–
	Weder noch	4 (1,9)	4 (1,9)	–	–
	Trifft eher zu	38 (17,8)	38 (17,8)	–	–
	Trifft voll und ganz zu	172 (80,4)	172 (80,4)	–	–
8. Für Studierende: Die Moulage hat mir dabei geholfen, die vorgestellten Hauteffloreszenzen besser zu verstehen und zuordnen zu können	Ja	5 (2,4)	5 (2,4)	–	–
	Nein	205 (97,6)	205 (97,6)	–	–
	Fehlend	4	4	–	–
