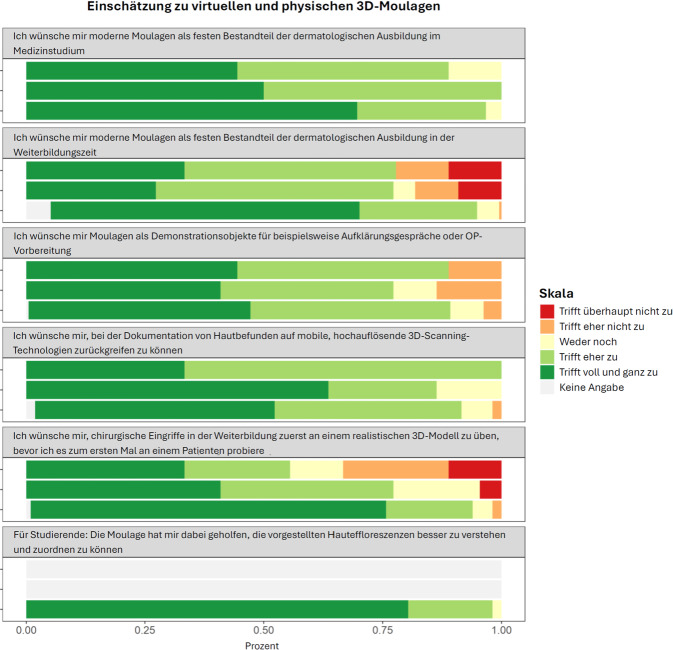# Erratum zu: Moulage 2.0: Eine Querschnittsstudie zu einem 3D-gedruckten Hautmodell in der dermatologischen Lehre und Weiterbildung

**DOI:** 10.1007/s00105-025-05590-1

**Published:** 2025-09-25

**Authors:** Alexander Schneller, Hannah Wecker, Michael Hindelang, Sandra Schuh, Julia Welzel, Alexander Zink

**Affiliations:** 1 Klinik und Poliklinik für Dermatologie und Allergologie am Biederstein, Fakultät für Medizin, Technische Universität München, München, Deutschland; 2 Klinik für Dermatologie und Allergologie, Universitätsklinikum Augsburg, Sauerbruchstr. 6, 86179 Augsburg, Deutschland; 3 Pettenkofer School of Public Health München, München, Deutschland; 4 Institut für Medizinische Informationsverarbeitung Biometrie und Epidemiologie (IBE), Ludwigs-Maximilians-Universität München, München, Deutschland; 5 Institut für Digitale Medizin, Universitätsklinikum Augsburg, Augsburg, Deutschland

**Erratum zu:**

**Dermatologie 2025**

10.1007/s00105-025-05579-w

Aufgrund eines Darstellungsfehlers war Abb. 6b in der ursprünglichen Datei seitlich beschnitten. Die Abbildung wurde im Originalartikel aktualisiert und liegt nun in vollständiger Form vor.

​